# Development and validation of immunogenic cell death-related signature for predicting the prognosis and immune landscape of uveal melanoma

**DOI:** 10.3389/fimmu.2022.1037128

**Published:** 2022-11-16

**Authors:** Yuanyuan Hu, Jiayang Cai, Meng Ye, Qianxue Mou, Bowen Zhao, Qian Sun, Xiaotong Lou, Hong Zhang, Yin Zhao

**Affiliations:** ^1^ Department of Ophthalmology, Tongji Hospital, Tongji Medical College, Huazhong University of Science and Technology, Wuhan, China; ^2^ Department of Neurosurgery, Renmin Hospital of Wuhan University, Wuhan, Hubei, China

**Keywords:** immunogenic cell death, uveal melanoma, prognosis, immune landscape, immunotherapy

## Abstract

**Introduction:**

Uveal melanoma (UM) is the most common primary intraocular malignant tumor in adults, and the main treatment for UM is currently surgery and plaque brachytherapy. UM is highly susceptible to metastasis, which eventually occurs in nearly half of all patients; once metastasis occurs, patients have a poor prognosis and the condition is difficult to treat. Therefore, the identification of new and effective UM biomarkers is vital for the application of therapeutic strategies. Immunogenic cell death (ICD) is a type of regulatory cell death that activates adaptive immune responses and generates long-term immunological memory. ICD can promote antitumor immunity, which may be a potential immunotherapeutic strategy for UM.

**Methods:**

The data of UM from the Cancer Genome Atlas (TCGA) was used as a training set and the data from Gene Expression Omnibus (GEO) was used as a validation set. To determine the expression pattern of ICD-related genes in UM, survival analysis and difference analysis was conducted. The ICD-related risk signature was constructed by employing the least absolute shrinkage and selection operator (LASSO) Cox regression. Subsequently, immune profile and somatic mutation analysis were performed. In addition, cell experiments were performed to verify the role of immunogenic cell death-related genes in UM.

**Results:**

In this study, we analyzed the relationship between ICD-related gene expression and UM patient prognosis, somatic mutations, and the tumor immune microenvironment. Importantly, we constructed a 5-gene ICD-related risk signature and confirmed it as a novel prognostic biomarker in UM patients. We found that the high-risk group had more immune cell infiltration and a worse prognosis than the low-risk group. In cellular experiments, we confirmed the high expression of FOXP3 inMUM2B andOCM-1A cell lines and that knockdown of FOXP3 markedly inhibited the proliferation of UM tumor cells.

**Discussion:**

ICD-related genes play a critical role in the tumor immune microenvironment. Our results may contribute to the development of effective immunotherapies.

## Introduction

Uveal melanoma (UM) is the most frequent primary intraocular malignancy in adults, although it is a rare disease (1). More than 90% of UM originates from uveal melanocytes that are located in the choroid, only 6% originates in the ciliary body, and 4% originates in the iris (1). At present, the available primary treatment for UM is radiation therapy and surgery (2). Plaque brachytherapy, the dominant globe salvaging method to control primary intraocular tumors (3, 4) also comes with some inevitable complications, including severe radiation retinopathy and visual loss (2). Despite good local therapy and control of the tumor, the mortality rate for UM remains high. A previous study showed that the 5-year and 15-year disease-related mortality rates for uveal melanoma patients were 31% and 40%, respectively (5). In addition, approximately half of the patients develop metastases, resulting in a poor prognosis, regardless of treatment for the primary tumor (1, 5, 6). Metastatic UM exhibits a poor response to chemotherapy or targeted therapy, and median survival in metastatic UM is about one year (1, 7). This mortality is partly due to a lack of understanding of the exact etiology and pathogenesis of UM. Therefore, the identification of new and effective UM biomarkers is vital for the application of therapeutic strategies.

Immunogenic cell death (ICD), a type of regulatory cell death (RCD), activates adaptive immune responses and generates long-term immunological memory (8, 9). ICD can be induced by a set of stimuli and antitumor therapies, including viral infection, chemotherapy, epigenetic modifiers, targeted anticancer agents, radiation therapy, and photodynamic therapy (9–11). IICD is typically accompanied by the release of numerous damage-associated molecular patterns (DAMPs), which can promote the recruitment and maturation of antigen-presenting cells and are associated with the initiation of adaptive immunity (9). Indeed, accumulating clinical evidence suggests that DAMPs have predictive value for immunotherapy response in cancer patients (12). Furthermore, previous studies have shown that ICD can evoke anticancer immune responses (13, 14). Notably, recent studies have demonstrated that lurbinectedin and belantamab mafodotin, two antitumor drugs with FDA approval for use in humans, are particularly efficient ICD inducers in cancer (15, 16). Therefore, it is important for cancer therapy to be able to induce ICD clinically. However, there is still a lack of sufficient evidence regarding the clinical application of ICD, particularly in the identification of ICD-related biomarkers.

Immunotherapy is developing rapidly and has emerged as a new treatment strategy for various cancers (17, 18). Immune checkpoint inhibitors (ICIs) have become one of the most promising modalities for fighting cancer (19). Programmed death 1 (PD-1)/programmed death ligand 1 (PD-L1) inhibitors have made breakthroughs in the treatment of melanoma (20), and their role in UM is under study. Specifically, a recent study demonstrated that ICD can evoke systemic antitumor immunity to inhibit metastasis in UM (21). ICD may be a potential immunotherapeutic strategy for UM. Consequently, it is of great importance to explore ICD-related biomarkers in UM and evaluate the possibilities of ICD, which may be practical for understanding the underlying pathogenesis of UM.

Here, we identified biomarkers associated with ICD, constructed an ICD-related prognostic signature, and confirmed its prognostic value for UM patients. Next, we evaluated the relationship between the immune microenvironment and the ICD-related signature, and predicted the response to immunotherapy in UMs.

## Methods and materials

### Datasets

The whole-genome RNA-seq expression data and related clinical data of 80 uveal melanoma patients were acquired from The Cancer Genome Atlas (TCGA) database, which was used as training sets. The RNA-seq transcriptome information of 57 patients, used as validation cohort, was downloaded from the Gene Expression Omnibus (GEO) database, accession number: GSE44299. In the study, the cases with no survival data were eliminated. For the normal set, 175 normal retinal pigmented epithelium (RPE)-choroid complex samples were retrieved from the GEO database, accession number: GSE29801. We used the “normalizeBetweenArrays” function in the R package “limma” to remove multiple batch effects in merging the RNA_seq data of TCGA and GEO. In addition, the experimental flow chart was drawn and displayed in the **Supplementary Figure 1**.

### Identification of ICD-related differentially expressed genes between uveal melanoma and normal tissues

We analyzed DEGs from ICD-Related genes from TCGA-LGG and GEO databases *via* the Wilcoxon test. P values less than 0.05 were considered significant. The co-expression network of these significant genes was constructed by GeneMANIA (http://www.genemania.org/).

### Consensus clustering

Consensus clustering was conducted by the R package “ConcensusClusterPlus” to identify molecular subtypes related to ICD. The ideal cluster numbers, between k = 2–10, were assessed 1,000 times. We used the “pheatmap” package in R to create a cluster map. In addition, the overall survival (OS) in two clusters were compared through Kaplan-Meier (KM) analysis with the “survminer” and “survival” packages in R software.

### Identification of DEGs in ICD-related clusters and functional enrichment analyses

The R package “limma” was utilized to assess the DEGs in two clusters. To rectify false-positive TCGA data, we set the filter condition that adjusted P values less than 0.05 and abs of logFC larger than 2.5. Followed, The R package ‘‘clusterProfiler’’ was employed to conduct Gene Ontology (GO) functional enrichment analyses and Kyoto Encyclopedia of Genes and Genomes (KEGG) pathways enrichment analyses. The q-value and p-value thresholds were less than 0.05 in the analysis. The Gene Set Enrichment Analysis (GSEA) was accomplished by using the R package ‘‘clusterProfiler’’.

### Somatic mutation analysis

The somatic mutation data of UM was downloaded from TCGA database. The “maftools” package of R software was used to calculate the Tumor mutational burden (TMB) of each sample and draw the waterfall plots.

### Depicting the relationship between molecular clusters and tumor microenvironment immune characteristics

To assess the stromal score, estimate score, immune score, and tumor purity of each UM sample, we employed the “estimate” package in R software. According to RNA profiles of UM samples from the TCGA database, we analyzed the expression levels of human leukocyte antigen (HLA) genes and Immune checkpoints (ICPs) with the “ggplot2” package. Wilcoxon test was performed to compare the difference in expression levels between the two groups, and a P value less than 0.05 was considered significant.

### Construction and validation of the ICD-related risk signature

Univariate Cox regression analysis was used to evaluate the prognostic value of ICD-Related genes in UM. Subsequently, to formulate a risk signature, the least absolute shrinkage and selection operator (LASSO) cox regression analysis was performed by using the genes with statistically significant, which can compute the regression coefficients of each gene. The calculation of risk scores was according to the following formula:


$$Risk\;Score\;=\sum_{1}^{n}k_{n}*A_{n}$$

where *An* denoted the expression value of ICD-related genes, *kn* denoted the regression coefficient of prognosis-related genes, and n is the number of ICD-Related genes. Patients with risk scores below the median were classified as the low-risk group, while those with risk scores above the median were classified as the high-risk group.

### Prognostic analysis and clinicopathological relevance of ICD-related risk signature

We conducted the KM analysis to assess the differences in the overall survival (OS) between the low- and high-risk groups through the R packages “survminer” and “survival”. The nomogram model, containing clinically relevant and prognostic factors, was constructed by the packages “rms,” “foreign,” and “survival” in R software. The Univariate Cox regression analysis was employed to identify Potential prognostic indicators and the multivariate Cox analysis was employed to confirm the independent prognostic factors in UM. Then, to assess prediction accuracy, we plotted the 1-, 3-, and 5-year receiver operating characteristic (ROC) curves and calculated the area under the ROC curves (AUCs), which can judge the accuracy of a diagnostic approach: low accuracy: 0.5< AUC-ROC ≤ 0.7, moderate accuracy: 0.7< AUC-ROC ≤ 0.9, and high accuracy: 0.9 < AUC-ROC ≤ 1 (22). In addition, we analyzed the relevance between the risk score and clinicopathological characteristics, including gender and grade, by Chi-square test. P values< 0.05 was considered significant.

### Gene set variation analysis and single-sample gene sets enrichment analysis

The Gene set variation analysis (GSVA) of KEGG pathway was performed among low- and high-risk groups in the training sets. Single-sample GSEA (ssGSEA) was utilized to calculate immune function scores. According to the ssGSEA scores, we assessed the activities and abundances of immune-related pathways and functions.

### Prediction of response to immunotherapy

We performed tumor immune dysfunction and exclusion (TIDE) analysis in order to evaluate immunotherapy response. TIDE (http://tide.dfci.harvard.edu/), as an analytic technique, could predict the immunotherapy response by using two major tumor immune evasion mechanisms: T cell dysfunction and T cell infiltration inhibited in tumors with low CTL levels. Next, the “ggplot2” package in R software was performed to make a graph.

### Forecasting of drug sensitivity

We compared drug sensitivity between ICD-high and ICD-low risk groups through the Genomics of Drug Sensitivity in Cancer (GDSC) database and analyzed the drug sensitivity of *ENTPD1*, *CASP8*, *LY96*, *FOXP3*, and *IL6 via* Gene Set Cancer Analysis (GSCA) (http://bioinfo.life.hust.edu.cn/GSCA/) and CellMiner (http://discover.nci.nih.gov/cellminer/) database.

### Antibodies and reagents

Anti-FOXP3 (A4953, ABclonal); Anti-β-Actin (AC004, ABclonal). Liposomal Transfection Reagent (40802ES02, Yeasen); a CCK-8 cell counting kit (40210ES10, Yeasen); Hoechst (40730ES03, Yeasen); RIPA buffer (Applygen Technologies, Beijing China); protease and phosphatase inhibitors (Boster Biologic Technology).

### Cell culture and transfection

ARPE-19 cells were a gift from Zhongshan Ophthalmic Center, Sun Yat-Sen University. MUM2B and OCM-1A cells cells were purchased from iCell Bioscience Inc. MUM2B cells were maintained in RPMI 1640 culture medium (Boster Biologic Technology) supplemented with 10% FBS (Gibco, CA, USA) at 37°C with 5% CO2. The other cell lines were maintained in DMEM (Boster Biologic Technology) containing 10% FBS at 37°C with 5% CO2. MUM2B and OCM-1A cells were transfected with siFOXP3 by using Liposomal Transfection Reagent according to the transfection protocol. Cells were harvested at 48 hours of transfection for further analysis. Negative control siRNA (sc-37007) and FOXP3 siRNA (sc-43569) were purchased from Santa Cruz Biotechnology Inc. Sequence (5′ to 3′) of siFOXP3: #1 5’-AAGCAGCGGACACTCAATGAG-3’; #2 5’-AATGAGATCTACCACTGGTTC-3’.

### Cell viability assay

To detect the cell viability activity, a CCK-8 cell counting kit (CCK-8) was utilized. According to the manufacturer’s instructions, MUM2B and OCM-1A cells were inoculated in 96-well plates with 5000 cells in each well and cultured in a 37°C containing 5% CO_2_. Cells were transfected 12 hours after inoculation, and 48 hours after transfection, CCK-8 reagent was added and incubated for 1 hour. A microplate reader was used to measure the OD value at 450 nm. The experiments were conducted in triplicate at least.

### EdU-DNA synthesis assay

A Cell-Light EdU Apollo567 *In Vitro* Kit (C10310–1, RiboBio, Guangzhou, China) was utilized to conduct the experiment of proliferating cells. Cells were transfected 12 hours after inoculation onto the crawl sheets, and then cultured for 48 hours after transfection for harvesting. Following the manufacturer’s instructions, cells were incubated with 50 μM EDU medium for 2 h, and then were fixed with 4% paraformaldehyde for 30 min. Followed the decolorization and membrane rupture, Hoechst and 1X Apollo staining reaction solution were added and incubated for 30 min. A fluorescence microscope (Olympus BX51, Japan) was used to capture the fluorescence of Hoechst and EdU. To count the cells, ImageJ software was used and 10 non-overlapping fields were selected to calculate the proportion of EdU-positive cells, which represented the percentage of Edu-stained positive cells to Hoechst-positive cells.

### Protein extraction and immunoblot analysis

The RIPA buffer supplemented with protease and phosphatase inhibitors was used to lysed cells. After lysis, the protein concentration was detected by the BCA method, and the lysis was mixed with loading buffer (Beyotime) and heated on a 95°C thermostat for 5 min. The equivalent amounts of samples were resolved by SDS-PAGE and transferred to polyvinylidene difluoride (PVDF) membranes (MilliporeSigma). Membranes were blocked with 5% non-fat dry milk in TBST and treated with primary antibody overnight at 4°C, and then incubated with the secondary antibody (Proteintech, Wuhan, China) for 2 hours.

### RNA isolation and real-time quantitative PCR

RNA was extracted from the cells by the Trizol method, and then the concentration of RNA was detected. The reverse-transcription (RT) reaction was performed with PrimeScript™RT reagent Kit (RR047A; Takara Biomedical Technology). The real-time quantitative PCR reaction was performed with TB GreenPremix Ex Taq (RR420A; Takara Biomedical Technology). All samples were repeated at least three times with blank controls. Using the 2−ΔΔCT method to calculate the relative gene expression with normalization against Gapdh levels.

Gapdh: forward 5′-GGAGTCCACTGGCGTCTTCA-3′,

reverse 5′- GTCATGAGTCCTTCCACGATACC-3′,

Foxp3: forward 5’-GAGAAGCTGAGTGCCATGCA-3’

reverse 5’-AGAGCCCTTGTCGGATGAT-3’.

### Statistical analysis

Unless specifically stated the experiments were repeated at least three times. The results were presented as mean ± S.D., and statistical analyses were performed in GraphPad Prism 7. One-way analysis of variance (ANOVA) was used to assess statistical significance among the experimental groups. P values less than 0.05 were considered significant.

## Results

### Expression profiles and clusters of ICD-related genes in UM

**Figure 1:**
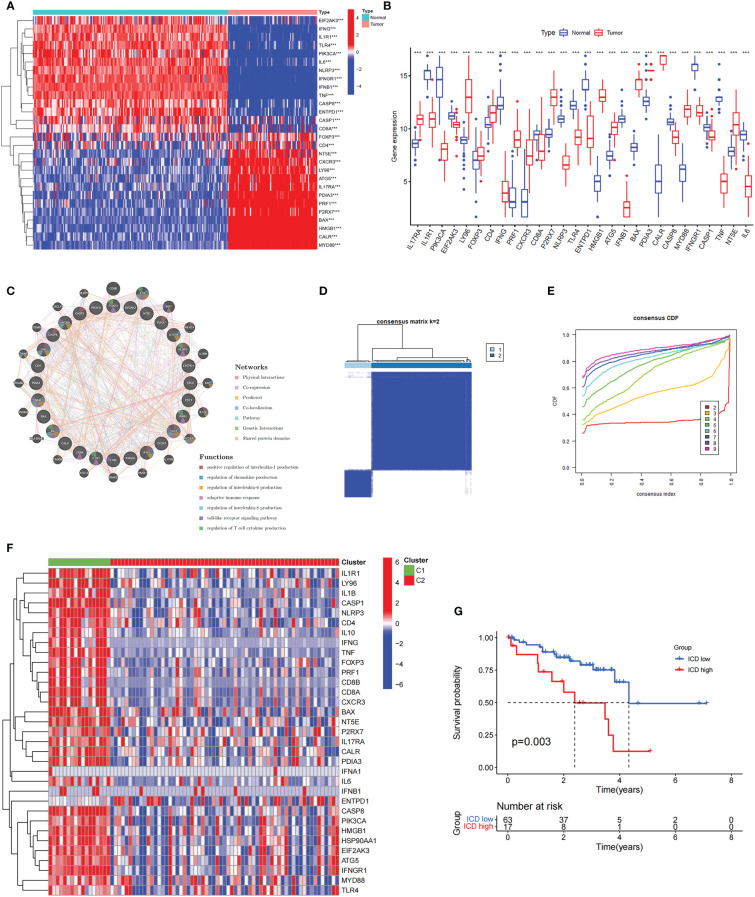
The expression profiles and the cluster of ICD-related genes in uveal melanoma. **(A, B)** The heatmap **(A)** and box plot **(B)** show that the expression patterns of 28 of the 34 ICD-related genes differed significantly between normal and UM samples in TCGA and GEO databases, ***P< 0.001. **(C)** Analysis of differentially expressed genes and their co-expressed genes by GeneMANIA. **(D)** Heatmap of consensus clustering solution (k = 2) for ICD-related genes in 80 UM samples. **(E)** The cumulative distribution function (CDF) curve of consensus clustering for k = 2 to 10. **(F)** Heatmap of ICD-related gene expressions in different subtypes. Green represents the high expression group and red represents the low expression group. **(G)** Kaplan–Meier curves of OS in the high and low ICD expression groups.

A summary of 34 ICD-related genes was reported by Abhishek et al. based on a large-scale meta-analysis (23). First, we investigated the expression profiles of ICD-related genes in normal and UM samples. We found that half of the ICD-related genes were highly expressed in UM, including *FOXP3, CD4, CXCR3, NT5E, ATG5, LY96, IL17RA, PDIA3, PRF1, P2RX7, HMGB1, BAX, CALR*, and *MYD88*, whereas the *CD8A, IL6, PIK3CA, EIF2AK3, NLRP3, TLR4, ENTPD1, IFNGR1, CASP1, TNF, IFNG, IL1R1, IFNB1*, and *CASP8* genes were underexpressed (**Figures 1A, B**). Subsequently, the coexpression network confirmed a strong coexpression correlation among these genes (**Figure 1C**).

To further explore the role of ICD-related genes in UM, we performed a recognition of ICD-related clusters in UM through consensus clustering (**Figures 1D, E**). As shown in **Figure 1D**, the TCGA cohort was grouped into two clusters, C1 and C2, and the heatmap revealed the differential expression of ICD-related genes in these two clusters (**Figure 1F**). Therefore, Cluster C1, showing high expressions of ICD-related genes, was defined as an ICD-high subtype. Conversely, Cluster C2, displaying low expression levels, was defined as an ICD-low subtype. Furthermore, survival analysis illustrated a better prognostic potential in the ICD-low subtype than in the ICD-high subtype (**Figure 1G**).

### Analyses of differentially expressed genes and functional enrichment in different ICD clusters

**Figure 2:**
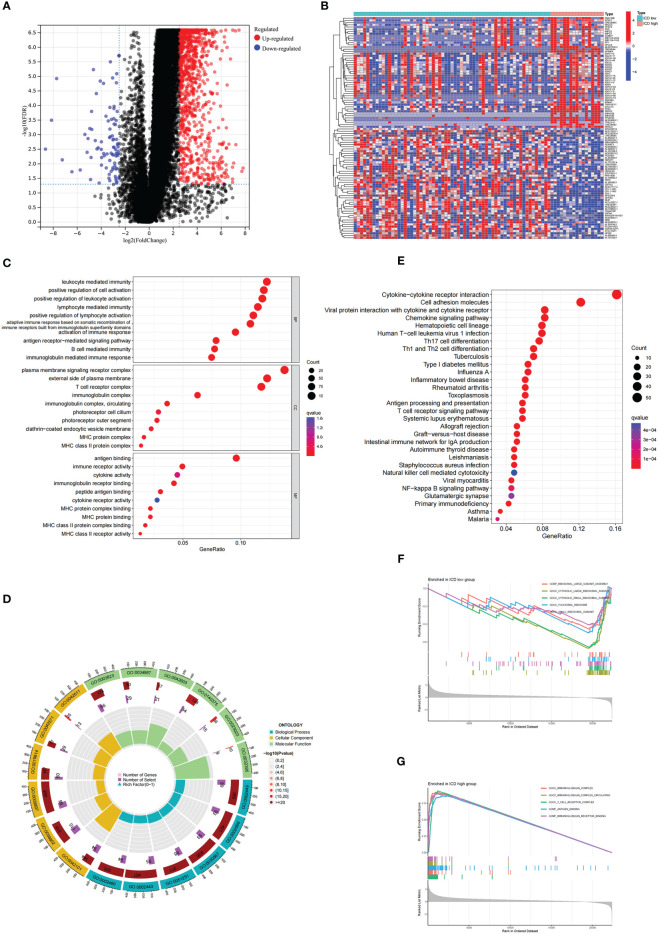
Analysis of the DEGs and functional enrichment analysis in different ICD clusters. **(A)** Volcano plot displays the DEGs between the high and low ICD expression groups in the TCGA cohort. **(B)** Heatmap of the DEGs expression between the high and low ICD expression groups. **(C, D)** The dots **(C)** and circle plots **(D)** of GO signaling pathway enrichment analysis. **(E)** The dots plot of KEGG signaling pathway enrichment analysis. The size of the dot represents gene count, and the color of the dot represents the q value. **(F, G)** GSEA analysis shows the underlying signal pathway between the low **(F)** and high **(G)** ICD expression groups. The top 5 signaling pathways were shown on the graph.

To understand the molecular mechanisms affecting prognosis, we analyzed the DEGs of ICD-high and ICD-low subtypes. We obtained a total of 1085 DEGs, which were utilized for functional enrichment analysis (**Figures 2A, B**). GO enrichment analysis showed that DEGs were enriched in activities associated with immunity, including leukocyte-mediated immunity, lymphocyte–mediated immunity, activation of immune response, and B-cell-mediated immunity (**Figures 2C, D**). Consistently, DEGs were significantly associated with Th17-cell differentiation, Th1 and Th2 cell differentiation, antigen processing and presentation, and natural killer cell-mediated cytotoxicity in KEGG enrichment analysis (**Figure 2E**). In addition, we performed GSEA between the ICD-high and ICD-low subtypes (**Figures 2F, G**). The results revealed that genes were principally enriched in the cytokine‒cytokine receptor interaction pathway in the ICD-high group. The above results suggested a strong correlation between DEGs and immunity in ICD-high and ICD-low subtypes.

### Somatic mutations and characterization of the immune landscape in different ICD clusters

**Figure 3:**
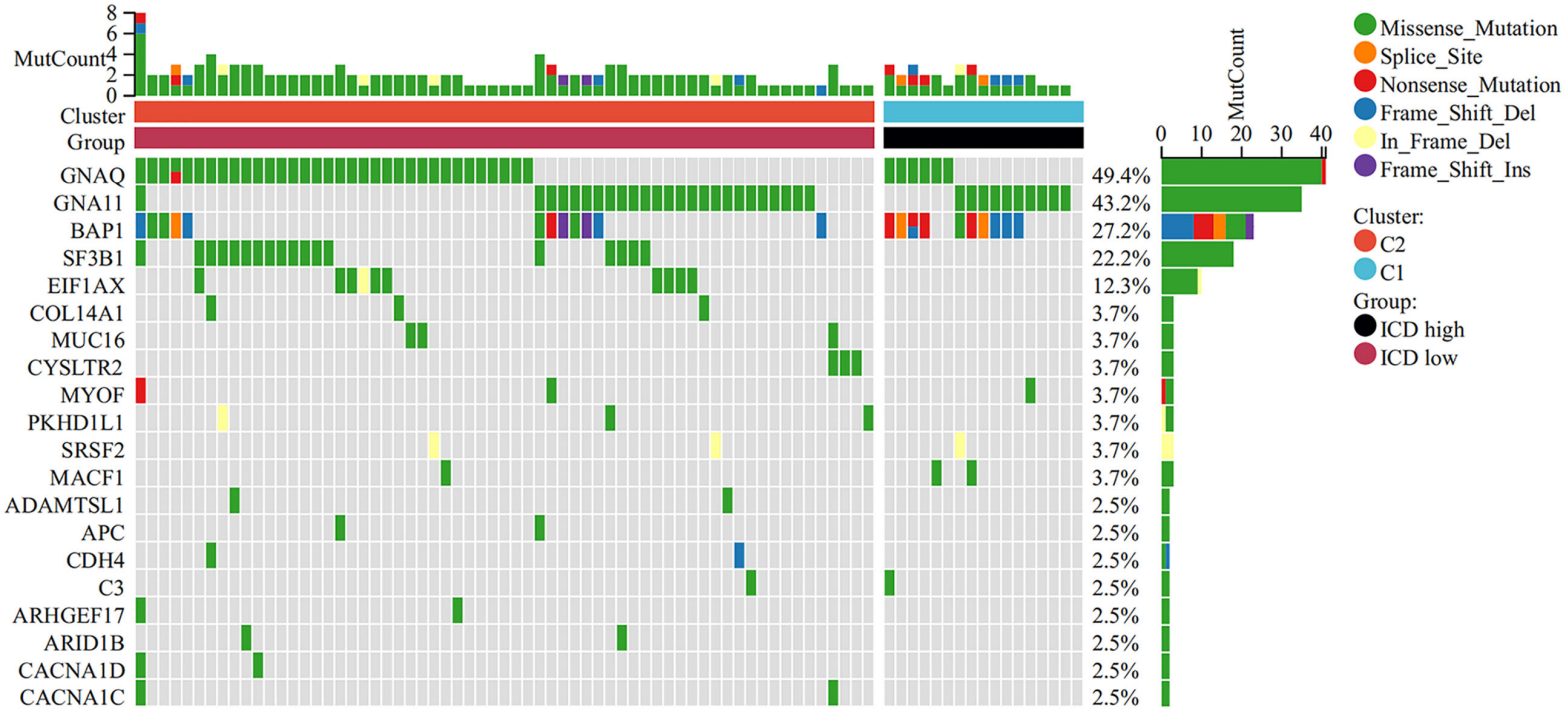
Comparison of somatic mutations in the high and low ICD expression groups. The top 20 most frequently mutated genes between the two groups were visualized in waterfall plots.

To better understand the genomic characteristics of ICD-high and ICD-low subtypes, we performed an analysis of somatic mutations. As shown in the waterfall plots, *GNA11*, *BAP1*, *MYOF*, and *MACF1* were the most frequent mutations in the ICD-high group, whereas the ICD-low group had a higher frequency of mutations in the *GNAQ*, *EIF1AX*, and *SF3B1* genes (**Figure 3**).

**Figure 4:**
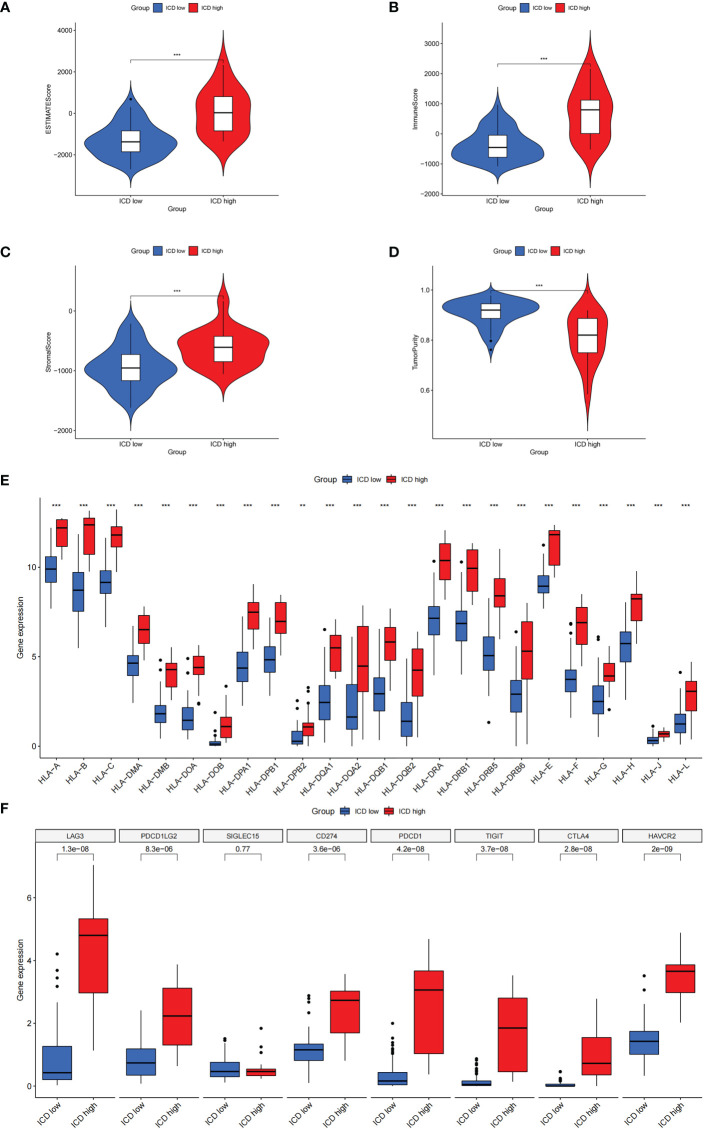
The characterization of immune landscape in different ICD clusters. **(A-D)** Violin plots of the stromal score, immune score, ESTIMATE score, and tumor purity between the high and low ICD expression groups. **(E, F)** Box plots of differential expressed immune checkpoints **(F)** and HLA genes **(E)** in two groups. **P< 0.01, ***P< 0.001.

There is growing evidence that ICD can cause anticancer immune responses (13, 14). Therefore, we explored the characterization of the immune landscape between ICD-high and ICD-low subtypes. We found that the ICD-high group had higher ESTIMATE scores, stromal scores, and immune scores, than those in the ICD-low group but had lower tumor purity (**Figures 4A–D**), which indicates that the levels of immune infiltration were higher. In addition, the roles of immune checkpoints (ICPs) and human leukocyte antigen (HLA) are essential in antitumor immune responses (24). We next probed their expression levels in different ICD clusters. It is obvious that ICPs and HLA expressions were significantly upregulated in the ICD-high group, including vital ICPs, such as *CD274*, *PDCD1*, and *CTLA4* (**Figures 4E, F**). In summary, these indicated that there is a strong link between the ICD-high subtype and an immune-hot phenotype, and the ICD-low subtype was associated with an immune-cold phenotype.

### Construction and verification of the ICD-related risk signature

**Figure 5:**
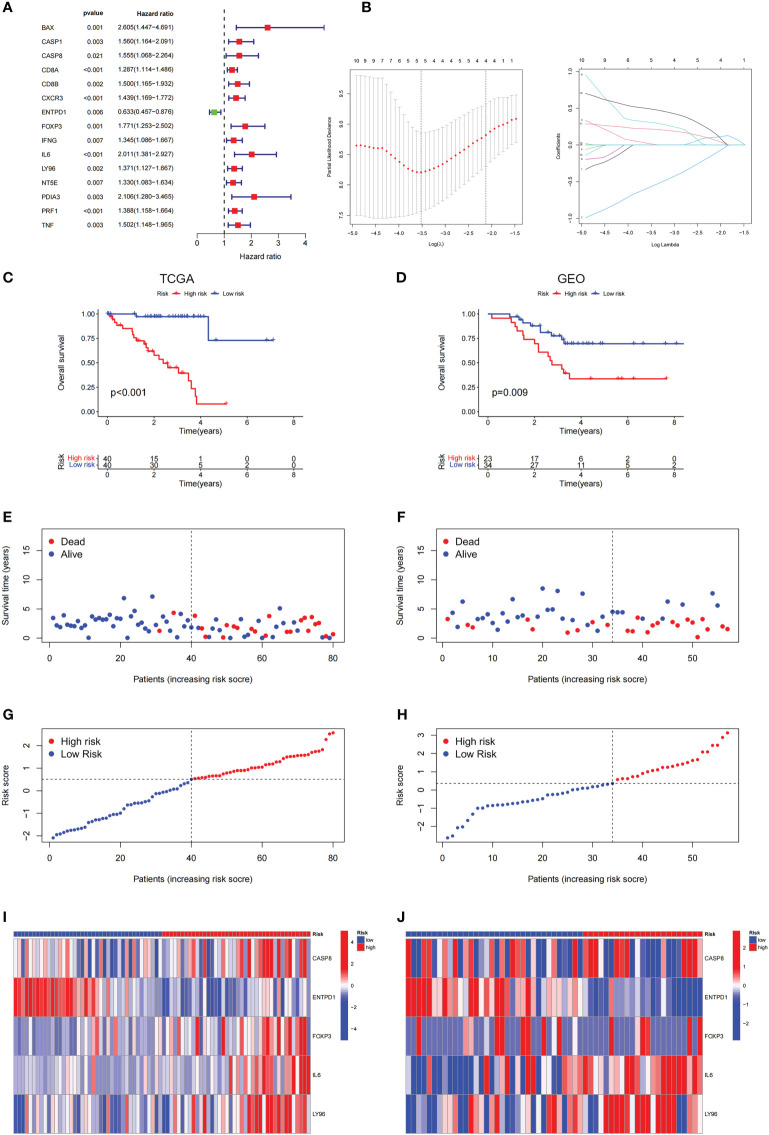
Construction and validation of the ICD-related risk signature. **(A)** The picture shows the overall survival (OS) forest plot obtained by univariate Cox analysis, which allows the assessment of the prognostic value of the ICD-related genes. **(B)** Lasso Cox analysis identified 5 ICD-related genes most associated with OS in the TCGA dataset. **(C, D)** Kaplan–Meier analysis of the prognostic significance of the risk model in TCGA and GEO databases. **(E, F)** The survival status of each patient in TCGA and GEO databases. **(G, H)** The distribution of risk scores in TCGA and GEO databases. **(I, J)** The heatmaps of prognostic 5 genes signature in the TCGA database and GEO databases.

To perform the LASSO regression analysis, we identified a total of 15 prognosis-related genes (p< 0.05) from ICD-related genes through univariate Cox analysis (**Figure 5A**). Following, 5 genes were selected for the optimal model in the LASSO regression analysis after validation (**Figure 5B**), which were sorted out in **Supplementary Table 1**. Furthermore, the KM analysis indicated that the high-risk score was linked to a poor prognosis in the TCGA cohort, GEO database further corroborated it (**Figures 5C, D**), which was consistent with the results of the progression-free survival (PFS) analysis (**Supplementary Figure 2**). Moreover, the distributions of survival status, risk score, and risk gene expression were plotted in TCGA and GEO databases, respectively (**Figures 5E–J**). The results above suggested that the risk score based on ICD-related risk signature might be a fine indicator for predicting the prognosis of patients with UM.

### The risk score could be an independent prognostic factor for predicting prognosis in UM patients

**Figure 6:**
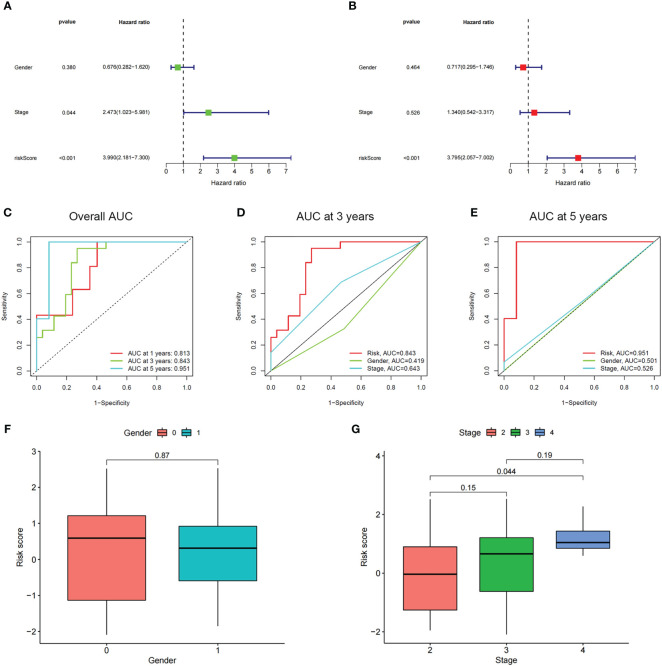
The prognostic value of the risk score and the association between risk score and clinicopathological factors. **(A)** Forest plot of the univariate Cox test to assess the correlation of risk scores and clinical factors with patient OS. **(B)** The forest plot of the multivariate Cox analysis identified independent prognostic factors associated with the OS of patients. **(C-E)** The ROC curve of risk score and clinical factors for predicting 1-, 3-, and 5-year OS in UM patients. **(F, G)** Distribution of ICD-related risk scores among UM patients stratified by gender and stage in TCGA database.

**Figure 7:**
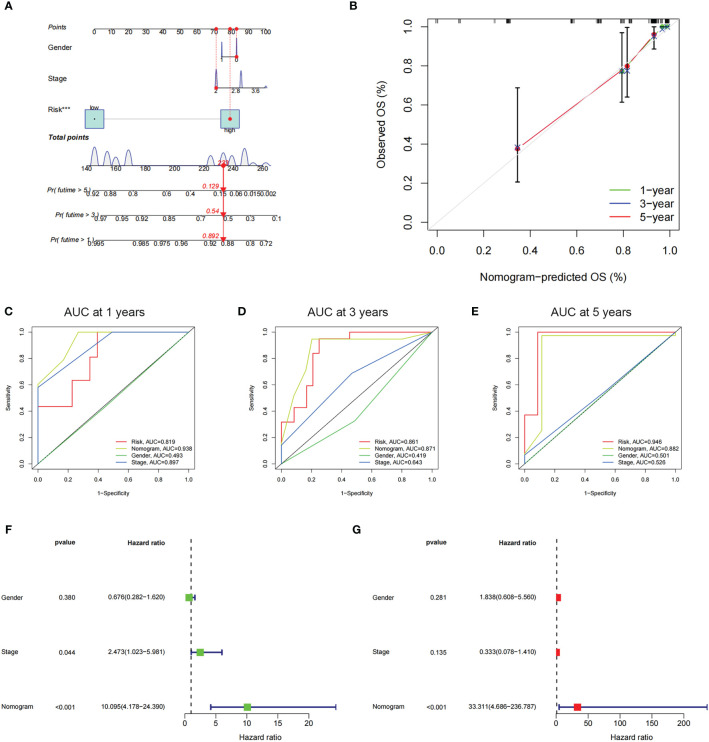
The prognostic value of risk score combined with clinicopathological features for OS of patients from the TCGA database. **(A)** The nomogram shows the OS of the UM patients from the TCGA database. **(B)** The nomogram’s calibration plots. The y-axis represents actual survival, whereas the x-axis represents nomogram-predicted survival. **(C-E)** ROC curve of risk scores and clinicopathological factors for predicting 1- **(C)**, 3- **(D)**, and 5-years **(E)** OS in UM patients. **(F, G)** The nomogram’s univariate and multivariate Cox regression analyses.

Through employing the univariate and multivariate COX analyses, we found that the risk score might be an independent factor for predicting the OS in UM patients (**Figures 6A, B**) and the area under the AUC-ROC curve for the risk score is larger than all other prognosis-related clinical factors, including gender and stage, in predicting 3 -, or 5-year survival (**Figures 6C–E**). Through exploring the relationship among risk scores and other clinical factors, we discovered that the risk score was associated with stage statistically, especially stage 2 and stage 4 (**Figures 6F, G**). To optimize the clinical application of the risk score model which is based on ICD-related genes, a nomogram comprising gender, grade, and risk score was constructed to predict 1-, 3-, and 5-year OS of UM patients (**Figures 7A, B**). ROC analyses showed that the nomogram has a higher sensitivity in predicting 1-, and 3-year OS of UM patients, while in predicting 5-year OS, the risk score has a better value (**Figures 7C–E**). Moreover, univariate and multivariate COX analyses showed that nomogram is also an independent prognostic factor in UM patients (**Figures 7F, G**). These results indicated that we could better predict the OS of UM patients by combining the nomogram and risk score.

### Analysis of DEGs, functional enrichment, and somatic mutations in different risk groups

**Figure 8:**
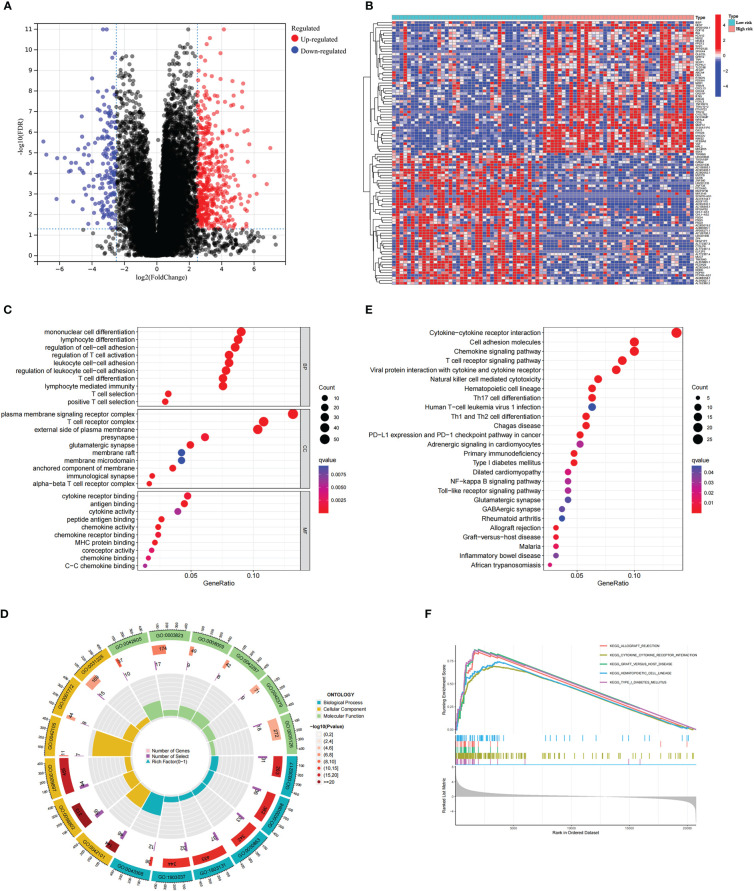
Analysis of the DEGs and functional enrichment analysis in different ICD risk scores. **(A)** Volcano plot displays the DEGs between the high and low ICD risk groups in the TCGA cohort. **(B)** Heatmap of the DEGs expression between the high and low ICD risk groups. **(C-E)** The GO **(C, D)** and KEGG **(E)** signaling pathway enrichment analysis. **(F)** GSEA analysis shows the underlying signal pathway between the high ICD risk group.

**Figure 9:**
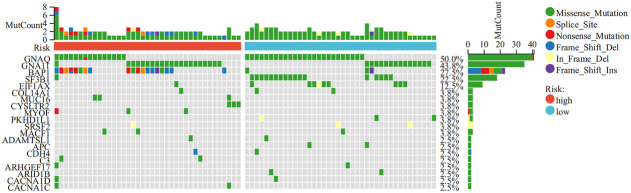
Comparison of somatic mutations in the high and low ICD risk groups. The top 20 most frequently mutated genes between the high and low ICD risk groups were visualized in waterfall plots.

Next, we conducted genetic differences analysis between ICD-high risk and ICD-low risk groups and obtained 450 upregulated and 166 down-regulated genes (**Figures 8A, B**). GO functional enrichment analysis showed that these genes are mainly involved in mononuclear cell differentiation, lymphocyte differentiation, regulation of leukocyte cell-cell adhesion, regulation of T cell activation, T cell receptor complex, immunological synapse, antigen binding, and MHC protein binding (**Figures 8C, D**). KEGG analysis showed that these gene enriched in T cell receptor signaling pathway, PD-L1 expression and PD-1 checkpoint pathway in cancer, Th1 and Th2 cell differentiation, Natural killer cell mediated cytotoxicity, Primary immunodeficiency and NF-kappa B signaling pathway (**Figure 8E**). We also performed GSEA in ICD-high risk group and the results were showed in **Figure 8F**. In addition, the analysis of somatic mutation in different risk groups was conducted and indicated that *GNA11* and *BAP1* were more likely to be mutated in high-risk group while *GNAQ*, *SF3B1*, *EIF1AX* were the most frequent mutations in low-risk group (**Figure 9**).

### Association between risk score and immune landscape

**Figure 10:**
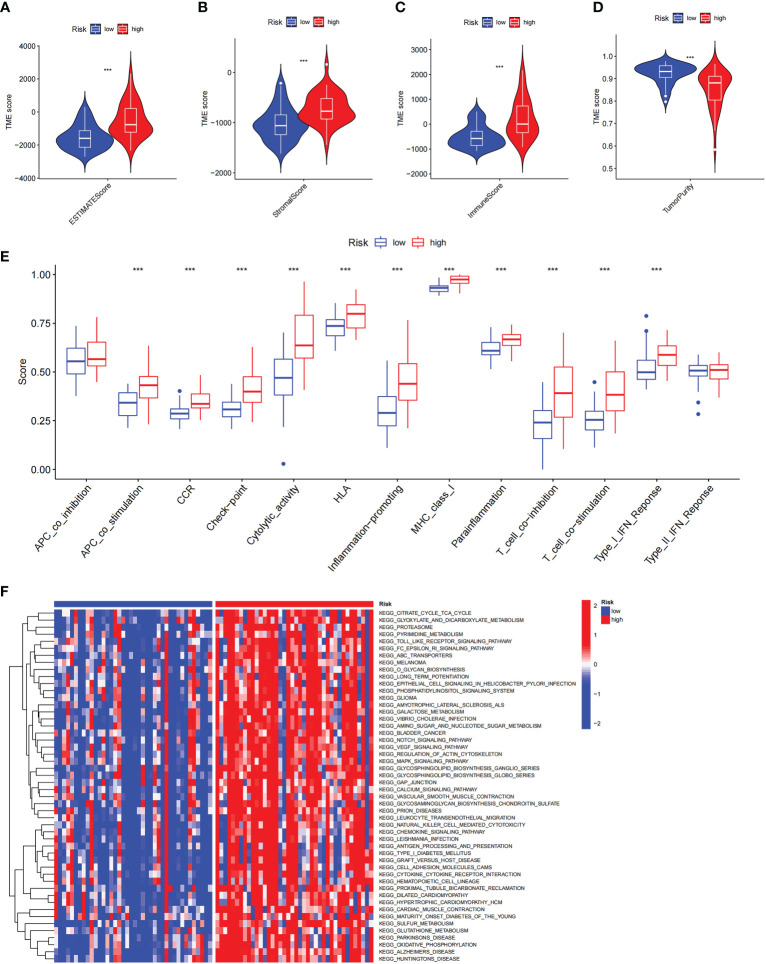
The immune landscape in the high and low ICD risk groups. **(A-D)** Violin plots of the stromal score, immune score, ESTIMATE score, and tumor purity between the high and low ICD risk groups. **(E)** Box plots show the differences in immune-related functions between the high and low ICD risk groups. **(F)** The heatmap of GSVA enrichment in two groups. ***P< 0.001.

**Figure 11:**
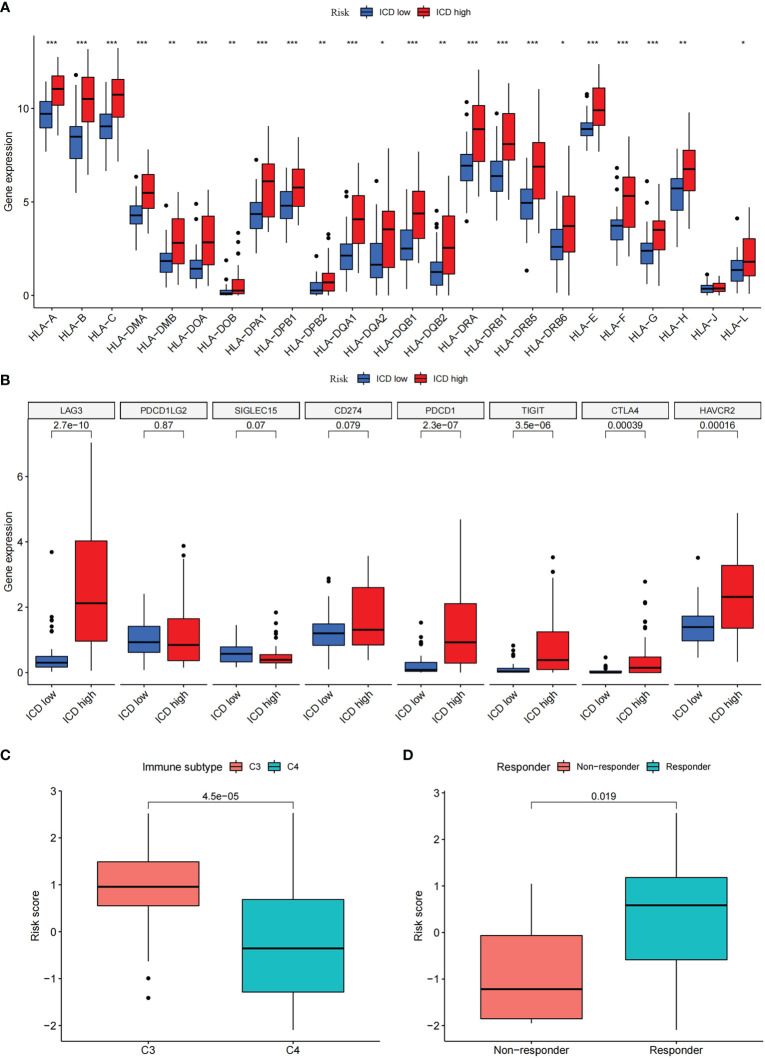
Differential expression of immune checkpoints and HLA genes. **(A, B)** Box plots of differential expressed immune checkpoints **(B)** and HLA genes **(A)** between the high and low ICD risk groups. **(C)** Box plots presents the association of ICD risk score with Immunotyping. **(D)** Box plot presents the links of ICD risk score with immunotherapy response. *P< 0.05, **P< 0.01, ***P< 0.001.

To explore the role of the risk score in immune infiltration, we investigated the ESTIMATE score between the ICD-high risk and ICD-low risk groups and found that the ICD-high risk group has a higher score in the stromal score, immune score, and ESTIMATE score, while has a lower tumor purity (**Figures 10A–D**). The results of immune-related functions showed that all functions including APC co-inhibition, APC co-stimulation, checkpoint, HLA, T cell co-inhibition, T cell co-stimulation, etc. are activated in the ICD-high risk group (**Figure 10E**). GSVA enrichment analysis showed that the ICD-high risk group has higher activity in immune-related pathways, such as natural killer cell-mediated cytotoxicity, antigen processing and presentation, leukocyte trans-endothelial migration, toll-like receptor signaling pathway, Notch signaling pathway, and other pathways (**Figure 10F**). Furthermore, we explored the expression of ICP genes and HLA genes in the different risk groups. Our results showed that all HLA genes have higher expression levels in the ICD-high risk group (**Figure 11A**). And most ICPs genes are highly expressed in the ICD-high group (**Figure 11B** and **Supplementary Figures 3A–C**). Consequently, the high-risk group had a strong correlation with immunization in our ICD-related risk profile model.

There are six types of immune subtypes, among which C3 represents inflammation and c4 represents lymphocyte depletion. As the picture shows that the risk score could distinguish between C3 and C4 immune subtypes (**Figure 11C**). Then, tumor immune dysfunction and exclusion (TIDE) was used to evaluate the predictive value of the ICD risk score in immunotherapy. In our results, ICD risk scores could distinguish between immunotherapy responders and non-responders, and the immunotherapy response group have a higher score, which indicated that patients with high ICD risk scores might benefit more from immunotherapy (**Figure 11D**).

### Identification of prognostic value of ICD-related risk gene and prediction of drug sensitivity

**Figure 12:**
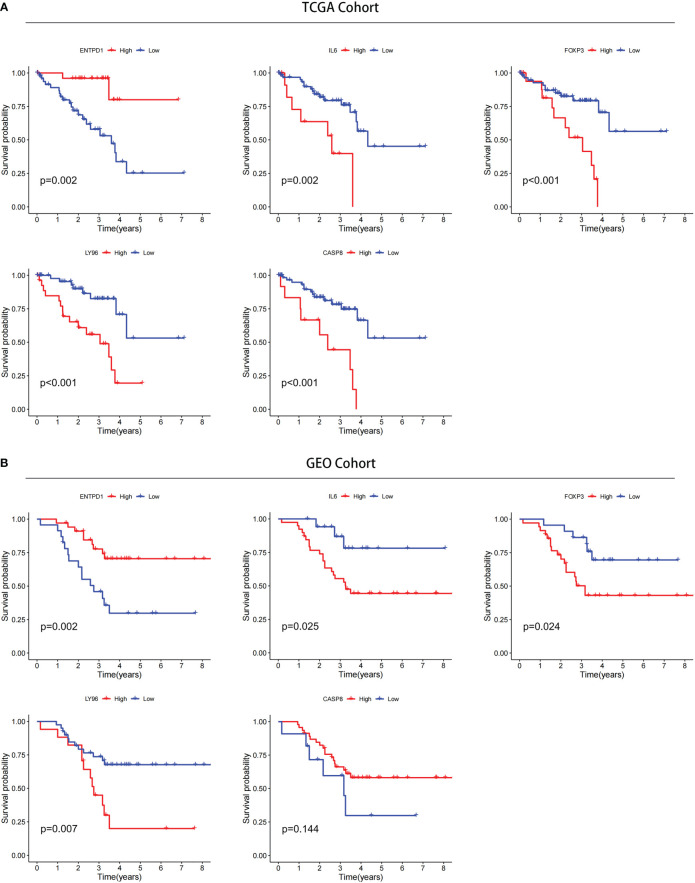
Confirmation of prognostic value of 5 ICD-related genes in TCGA and GEO databases. **(A)** Kaplan–Meier analysis of 5 ICD-related risk genes for patients in TCGA. **(B)** Kaplan–Meier analysis of 5 ICD-related risk genes for patients in GEO databases.

The prognostic value of 5 ICD-related genes involved in the risk model was explored. Our survival analysis results showed that patients with low expression of ENTPD1 have a poor prognosis, while patients with low expression IL6, FOXP3, and LY96 have a better prognosis both in TCGA and CGGA databases (**Figures 12A, B**). In addition, patients with CASP8 high expression have a poor prognosis in the TCGA database, but there was no significant difference in the GEO database. In addition, patients with CASP8 high expression have a poor prognosis in the TCGA database, but there was no significant difference in the GEO database.

Following this, we predicted the drug sensitivity by the GDSC database in different risk groups. we showed the top 12 drugs and found that most drugs were more sensitive to the high-risk group of patients (**Supplementary Figure 4**). In addition, utilizing the 5 ICD-related genes, we explored the drug sensitivity in pan-cancer in CellMiner and GSCA database (**Supplementary Figure 5** and **Supplementary Figure 6**).

### Experimental validation analysis

**Figure 13:**
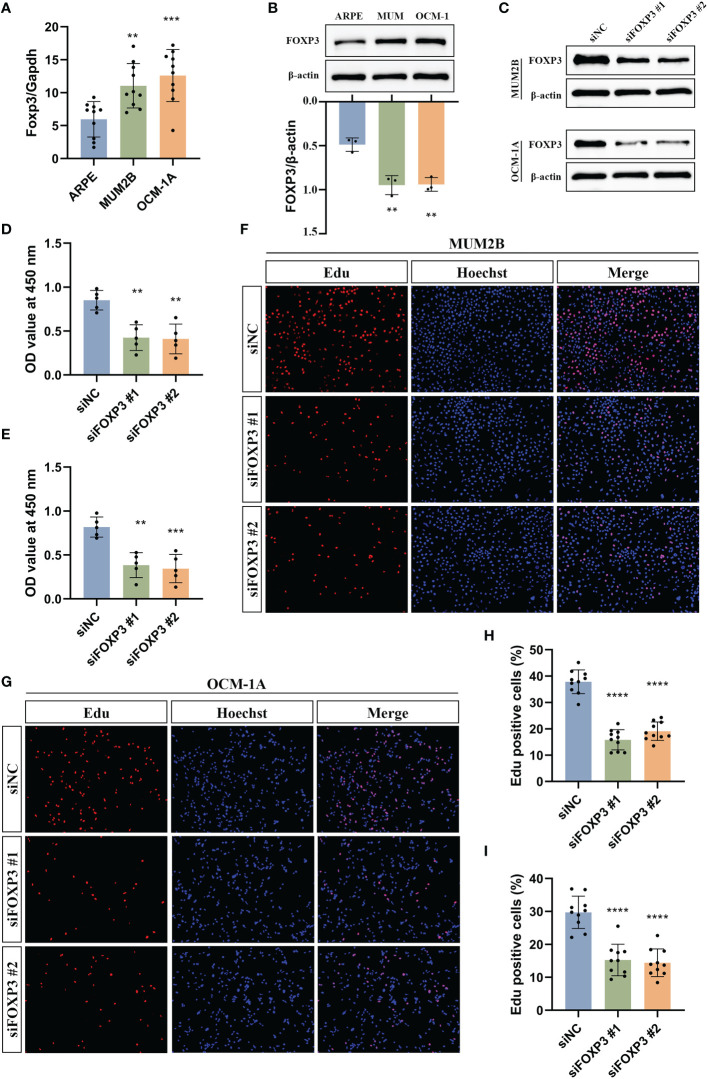
Experimental validation of the effect of FOXP3 on uveal melanoma growth. **(A)** The mRNA levels of Foxp3 in ARPE-19, MUM2B, and OCM-1A cells. **(B)** The protein levels of FOXP3 in ARPE-19, MUM2B, and OCM-1A cells. **(C)** Immunoblotting shows the knockdown efficiency of siRNA in MUM2B and OCM-1A cells. **(D, E)** CCK-8 assays were used to measure the viability of MUM2B **(D)** and OCM-1A **(E)** cells after the knockdown of FOXP3. **(F, G)** The assessment of cell proliferation by using the EdU-DNA synthesis assays following the knockdown of FOXP3. **(H)** The percentage of Edu-positive cells in **(F)** was displayed. **(I)** The percentage of Edu positive cells in **(G)** was displayed. **P< 0.01, ***P< 0.001, ****P< 0.0001.

The expression of FOXP3 has been reported to be associated with poor prognosis in some tumors (25–28), and FOXP3 can promote tumor growth in non-small cell lung cancer (25). However, the function of FOXP3 in UM has not been investigated. Thus, we selected FOXP3 to further explore potential cellular functions. First, we examined the mRNA expression levels of Foxp3 in ARPE-19 cells and uveal melanoma cells, including MUM2B and OCM-1A cells. The results showed that the mRNA level of Foxp3 was higher in uveal melanoma cells than that of ARPE-19 cells (**Figure 13A**). Consistently, the protein levels of FOXP3 were significantly increased in uveal melanoma cells (**Figure 13B**). Next, FOXP3 knockdown was performed by transfecting cells with siRNA (**Figure 13C**). CCK-8 assays showed that a significant decrease in cell proliferation was observed in both MUM2B and OCM-1A cells after FOXP3 knockdown (**Figures 13D, E**). In addition, we conducted EdU-DNA synthesis assays in these two cell lines (**Figures 13F, G**). The number of positive cells in the knockdown groups was distinctly reduced compared with that in the control group (**Figures 13H, I**). These results imply that FOXP3 is involved in tumor growth in UM.

## Discussion

In conclusion, we clarified the expression patterns of ICD-related genes in normal and uveal melanoma tissues and analyzed the relationship between ICD-related gene expression and UM patient prognosis, somatic mutations, and the tumor immune microenvironment. Importantly, we constructed a 5-gene ICD-related risk signature and demonstrated that it is a novel prognostic biomarker in UM patients. Based on the risk score, compared with the low-risk group, the high-risk group had a worse prognosis, more immune cell infiltration, and higher expressions of ICPs and HLA genes. In addition, we explored the relationship between risk scores and drug sensitivity. In cellular experiments, we confirmed the high expression of FOXP3 in MUM2B and OCM-1A cell lines and that knockdown of FOXP3 markedly inhibited the proliferation of UM tumor cells.

ICD can release danger signals or DAMPs to trigger adaptive immunological responses (8, 9). Several studies have revealed that chemotherapeutic agents and radiotherapy can induce ICD in tumor cells and produce a durable antitumor immune response (9, 29, 30). In the treatment of malignant tumors, immunogenic therapy combined with novel immunotherapy regimens holds great promise (31–33). However, ICD research in UM is still limited. In our study, we determined the differential expression patterns of ICD-related genes in normal and tumor tissues, and we divided tumor patients into ICD-high expression and ICD-low expression groups by consensus clustering based on the expression of these genes. It was reported that ICD can stimulate transformation of a “cold” immune environment into a “hot” immune environment and thus improve the response rate of patients to treatment (31). Here, we explored the tumor immune microenvironment in the two subtypes. The results showed that the high ICD expression group was correlated with the immune “hot” phenotype, and the low ICD expression group was associated with the immune “cold” phenotype.

Focal radiation therapy, including proton-beam radiation therapy, is a commonly used treatment modality to rescue the eye and can achieve high tumor control rates in over 90% of patients (2). Whereas primary UM can get local control, 50% of UM will eventually metastasize (6). Both uveal melanoma and cutaneous melanoma originate from melanocytes, but there are significant differences in their pathological features and treatment outcomes (1, 34). Immunotherapy has benefited most patients with cutaneous melanoma but has failed to achieve satisfactory results in the treatment of UM (35). A recent study found that ICD improved T-cell responses to different tumors, further enhancing the antitumor immunity of immune checkpoint inhibitors (36). Kim S et al. reported that combined treatment with ICD inducers and rock inhibitors enhanced the therapeutic effect of PD-1 blockers in mice with highly aggressive cold B16F10 tumors, targeting the primary tumor and inducing systemic antitumor immunity to inhibit metastasis (21). Epigenetic therapies can increase the immunogenicity of cancer cells (37), and the results of a phase II clinical trial by Ny L et al. indicated that the combination of epigenetic and immunotherapy resulted in tumor regression in a small percentage of patients with metastatic UM (38). Therefore, the identification of ICD-related biomarkers may allow patients to benefit from immunotherapy. Here, we constructed and validated a risk signature based on 5 ICD-related genes. We found that the risk signature had better prognostic power as an independent prognostic factor than other clinical factors. Consistently, the risk scores increased significantly with increasing tumor stage, suggesting that the risk score can distinguish tumor malignancy and correlate with tumor progression.

The genetic analysis of tumor samples in UM can help predict metastatic risk and manage patients. Monosomy 3 (M3) and *BAP1* gene inactivation are strongly associated with poor prognosis (39), and patients with *BAP1* inactivation are at high risk of metastasis (40, 41). Among the four subtypes identified by Robert son AG et al., *EIF1AX* or *SF3B1* alterations were associated with a lower risk of metastasis and a better prognosis (41). Although there is no conclusive evidence that alterations in the *GNAQ* and *GNA11* genes are relevant to UM prognosis and metastasis (39), a recent study reported a poorer prognosis for those with *GNA11* mutations (42). In our results, the frequencies of GN*A11* and *BAP1* gene alterations were highest in the ICD high expression and high-risk groups, indicating a poor prognosis. Consistently, *GNAQ*, *EIF1AX*, and *SF3B1* mutations were more common in the ICD low expression and low-risk groups and were associated with a better prognosis.

The eye is an immune-privileged organ giving a growth advantage to primary UM, and immune privilege may be responsible for immune evasion and systemic metastasis (2, 6). Given the immunosuppressive microenvironment of UM (6), it is important to understand the link between risk scores and immunological characteristics of UM samples. Our study identified a clear difference in immune cell infiltration among high- and low-risk groups. Immune cell infiltration in UM may be positively associated with poorer prognosis and metastatic death (43, 44). Our analysis revealed higher ESTIMATE scores and immune scores but lower tumor purity in the high-risk group, suggesting a poorer prognosis in the high-risk group. Subsequently, we demonstrated high expression of ICPs and HLA genes in the high-risk group and found a significant association between risk score and immunotherapy response. The expressions of ICPs and HLA genes are a potential factor for tumor immune escape (24). The potential association of the ICD-related risk signature with the tumor immune landscape established in this study may provide a prospective research direction for solid cancer immunotherapy.

In summary, ICD-related genes play a critical role in the tumor immune microenvironment. In our study, the construction of the ICD-related gene risk model predicted overall survival and response to immunotherapy in UM patients. Our results may contribute to the development of effective immunotherapies.

## Data availability statement

The datasets presented in this study can be found in online repositories. The names of the repository/repositories and accession number(s) can be found in the article/**Supplementary material**.

## Author contributions

YZ, HZ and YYH designed the research. JYC and YYH downloaded and analyzed the data. JYC, YYH, MY, BWZ, QS, XTL and QXM wrote the paper. All authors contributed to the article and approved the submitted version.

## Funding

The current study was supported by funding from the National Natural Science Foundation of China Grant no. 82070965 (to HZ) and Tongji Hospital (HUST) Foundation for Excellent Young Scientist Grant No. 2020YQ18 (to YZ).

## Conflict of interest

The authors declare that the research was conducted in the absence of any commercial or financial relationships that could be construed as a potential conflict of interest.

## Publisher’s note

All claims expressed in this article are solely those of the authors and do not necessarily represent those of their affiliated organizations, or those of the publisher, the editors and the reviewers. Any product that may be evaluated in this article, or claim that may be made by its manufacturer, is not guaranteed or endorsed by the publisher.

## Supplementary material

Supplementary Table 1 Genes and corresponding coefficients in ICD-related risk signature.

Supplementary Figure 1 The experimental flow chart.

Supplementary Figure 2 The progression-free survival (PFS) analysis between the high and low-risk groups.

Supplementary Figure 3 Differential expression of genes. These immune checkpoint genes with a fraction less than 10 were shown in **(A)**. These immune checkpoint genes with a fraction less than 100 and more than 10 were shown in **(B)**. These immune checkpoint genes with a fraction more than 100 were shown in **(C)**.

Supplementary Figure 4 The drug sensitivity comparison through the GDSC database between ICD-high and ICD-low risk groups.

Supplementary Figure 5 The correlation between the expression level of ENTPD1, CASP8, LY96, FOXP3, and IL6 and drug sensitivity in pan-cancer through the CellMiner database.

Supplementary Figure 6 The correlation between the expression level of ENTPD1, CASP8, LY96, FOXP3, and IL6 and drug sensitivity in pan-cancer through the GSCA database.
