# Supplementary material for: Development and validation of immunogenic cell death-related signature for predicting the prognosis and immune landscape of uveal melanoma
Source: Front Immunol. 2022 Nov 16;13:1037128. doi: 10.3389/fimmu.2022.1037128 (PMC9709208; doi:10.3389/fimmu.2022.1037128)
Supplement: Supplementary Table 1 — Genes and corresponding coefficients in ICD-related risk signature. [file Table_1.docx]

Supplementary Table 1. Genes and corresponding coefficients in ICD-related risk signature.

| Gene | Coefficient |
| --- | --- |
| CASP8 | 0.0163425975951479 |
| ENTPD1 | -0.574260300408961 |
| FOXP3 | 0.309512024606974 |
| IL6 | 0.49137706698673 |
| LY96 | 0.216847443498912 |
