# Supplementary figures and images for: Development and validation of immunogenic cell death-related signature for predicting the prognosis and immune landscape of uveal melanoma
Source: Front Immunol. 2022 Nov 16;13:1037128. doi: 10.3389/fimmu.2022.1037128 (PMC9709208; doi:10.3389/fimmu.2022.1037128)

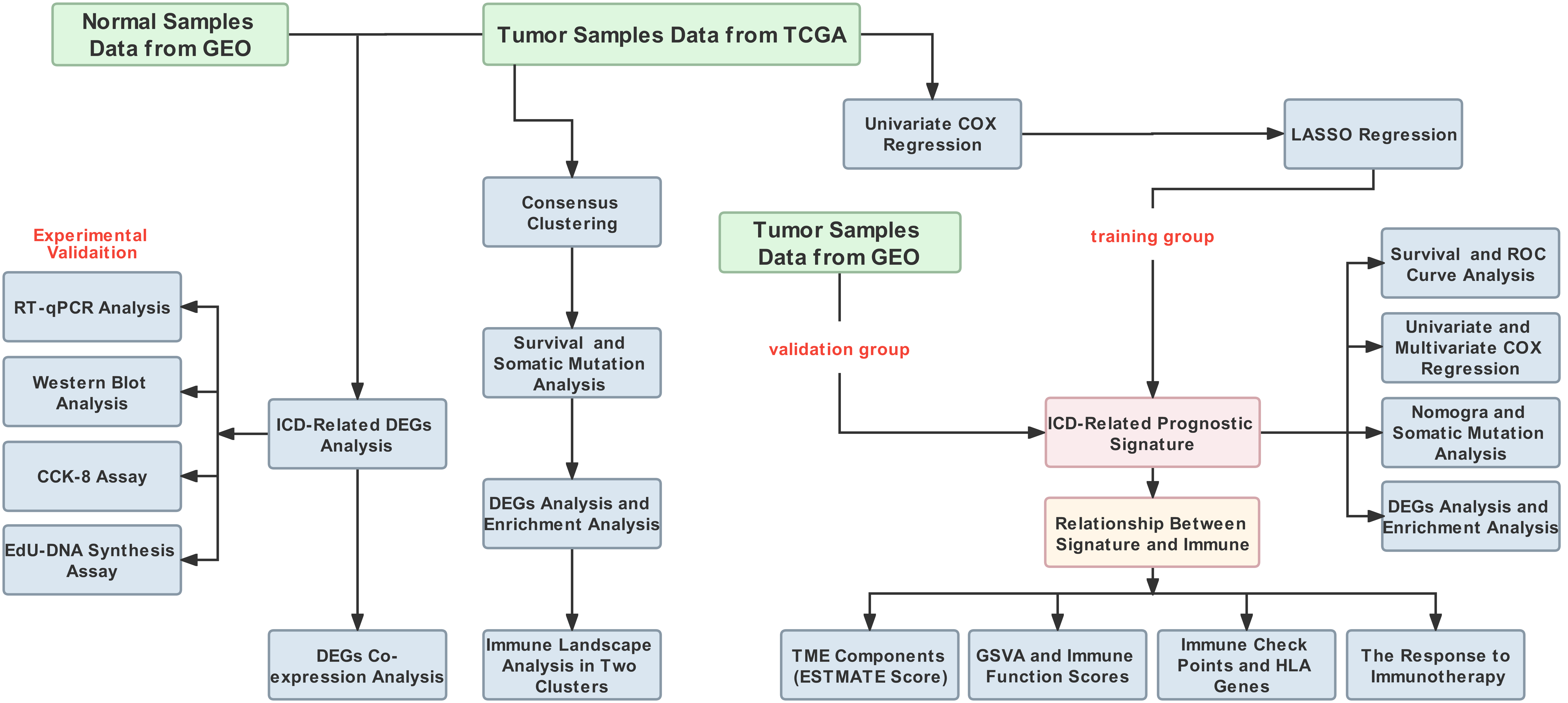

Supplement: Supplementary Figure 1 — The experimental flow chart. [file Image_1.tif]

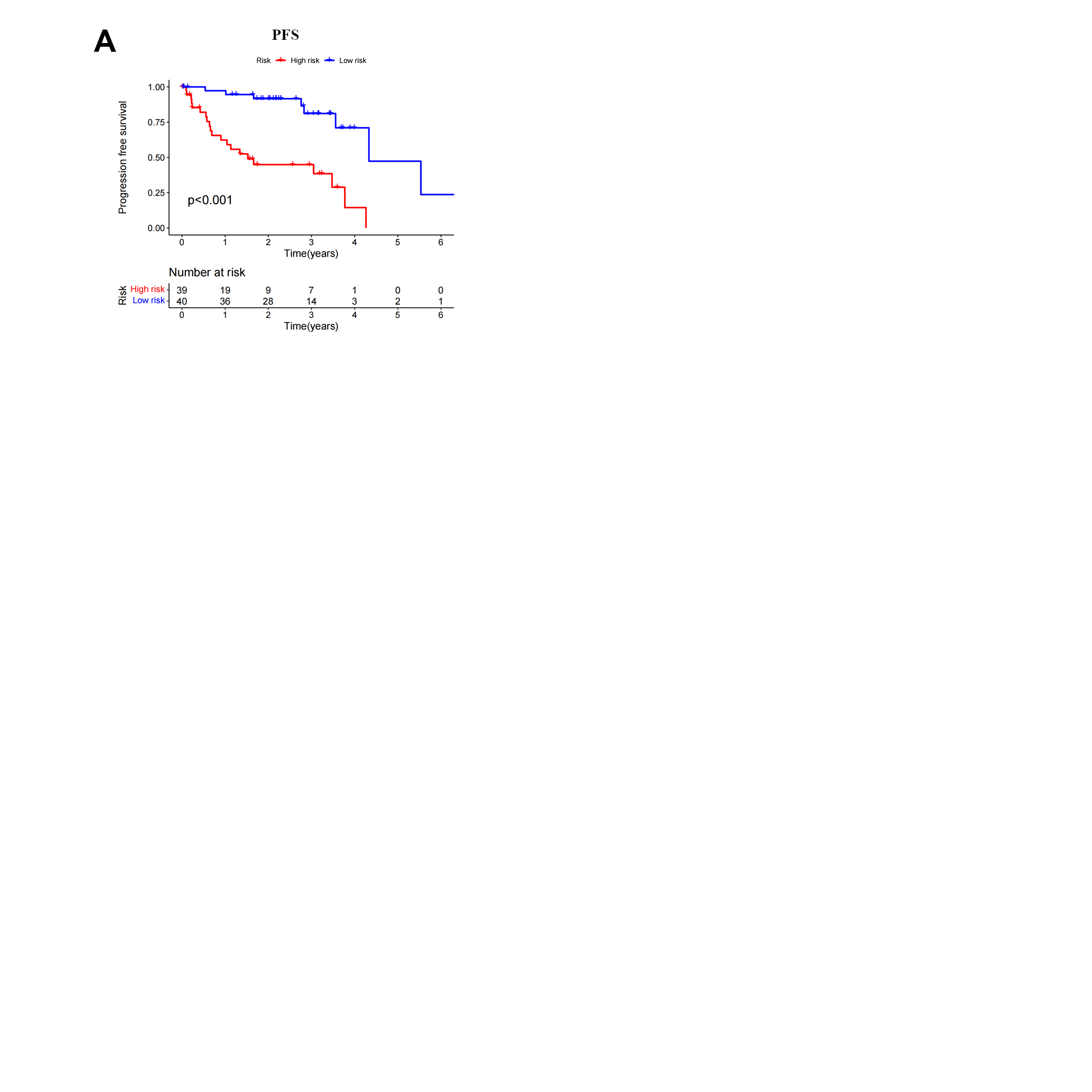

Supplement: Supplementary Figure 2 — The progression-free survival (PFS) analysis between the high and low-risk groups. [file Image_2.tif]

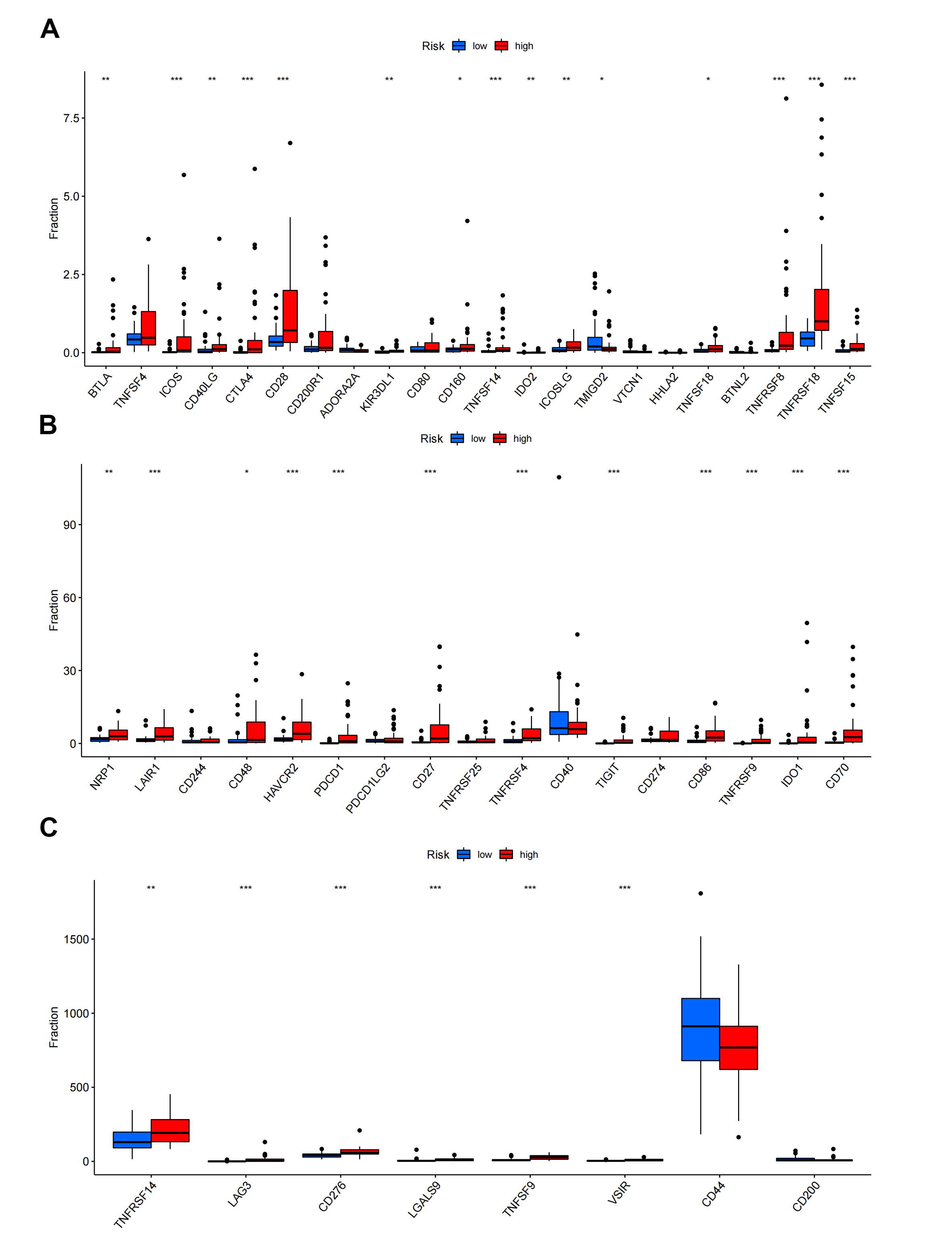

Supplement: Supplementary Figure 3 — Differential expression of genes. These immune checkpoint genes with a fraction less than 10 were shown in (A). These immune checkpoint genes with a fraction less than 100 and more than 10 were shown in (B). These immune checkpoint genes with a fraction more than 100 were shown in (C). [file Image_3.tif]

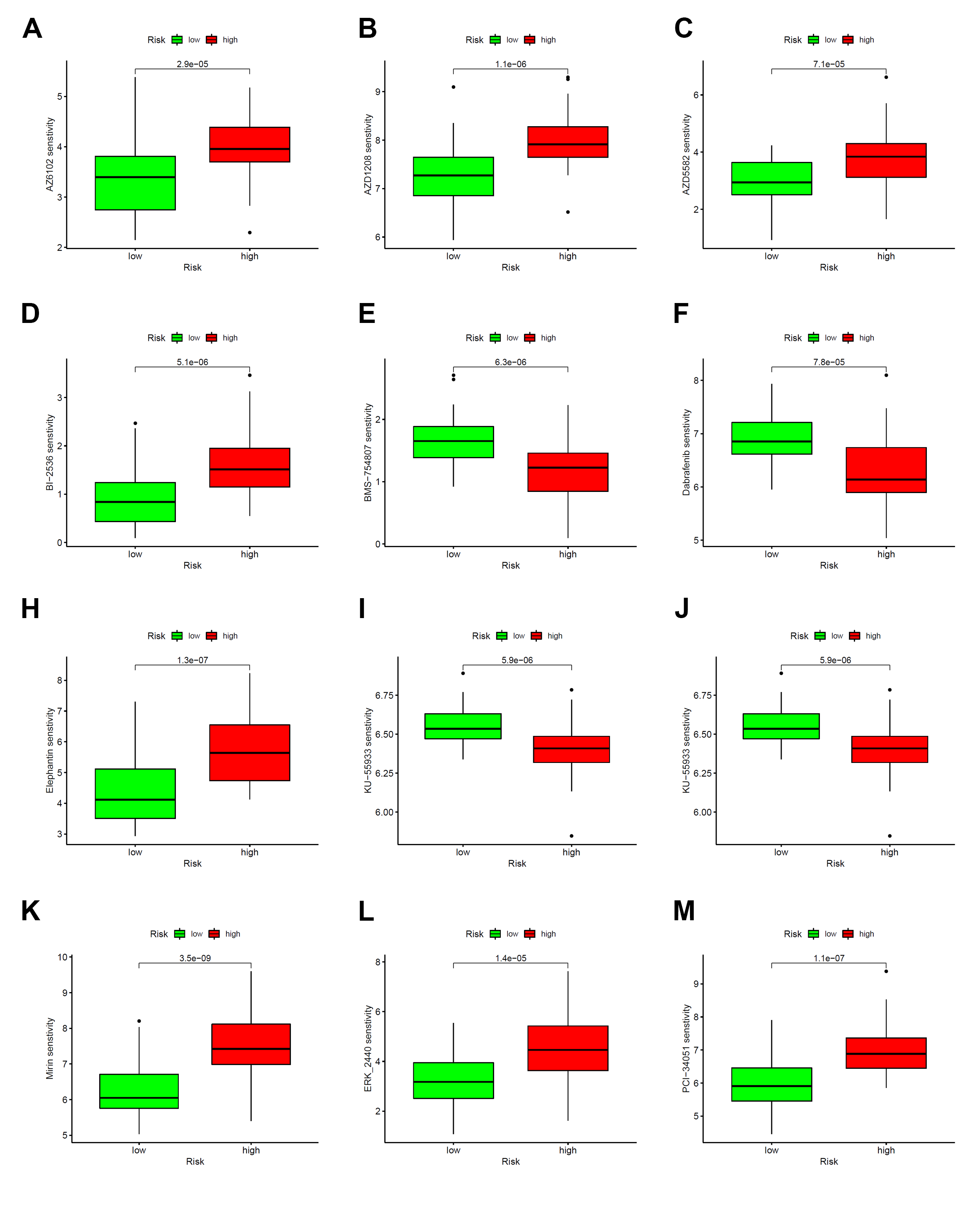

Supplement: Supplementary Figure 4 — The drug sensitivity comparison through the GDSC database between ICD-high and ICD-low risk groups. [file Image_4.tif]

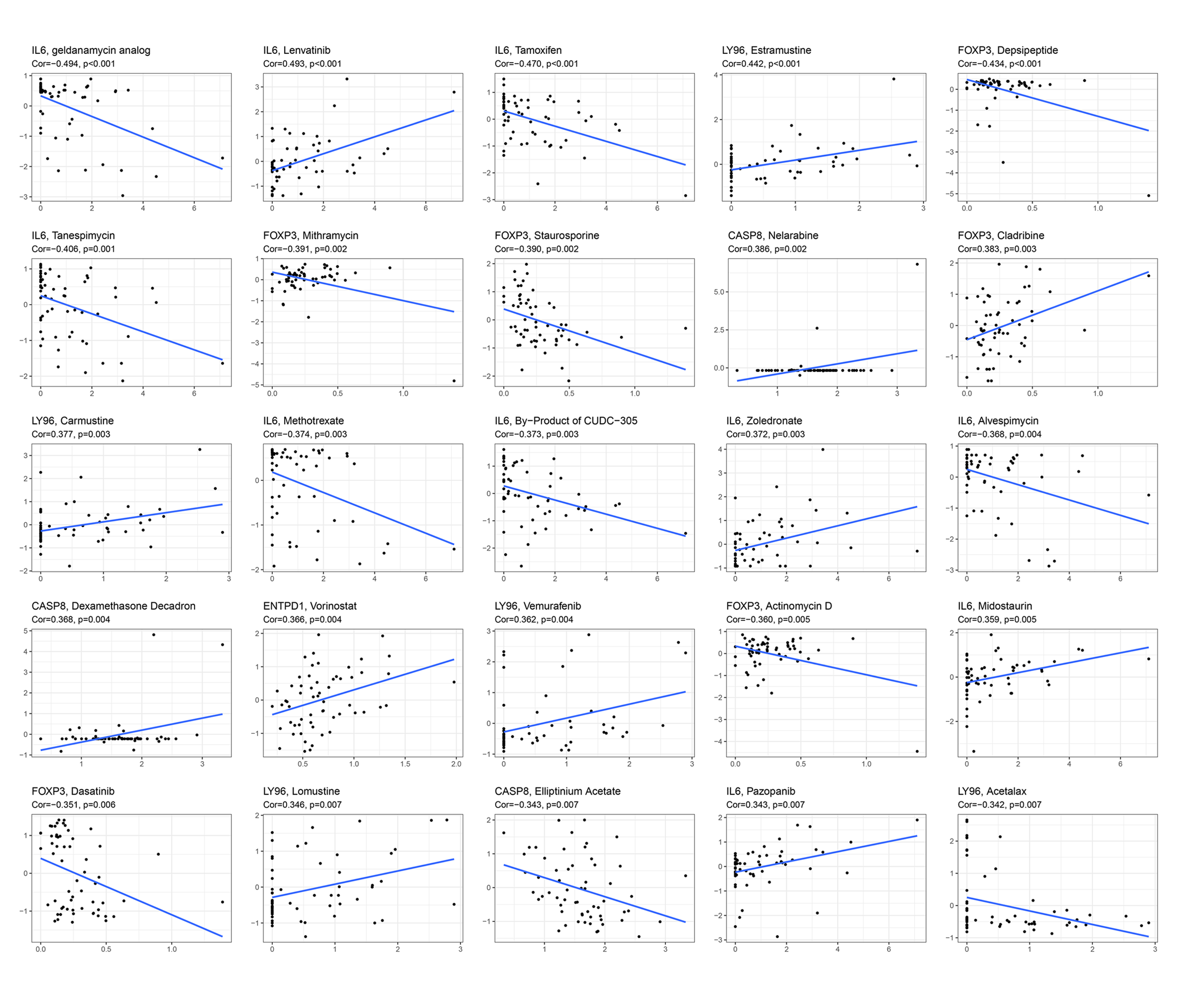

Supplement: Supplementary Figure 5 — The correlation between the expression level of ENTPD1, CASP8, LY96, FOXP3, and IL6 and drug sensitivity in pan-cancer through the CellMiner database. [file Image_5.tif]

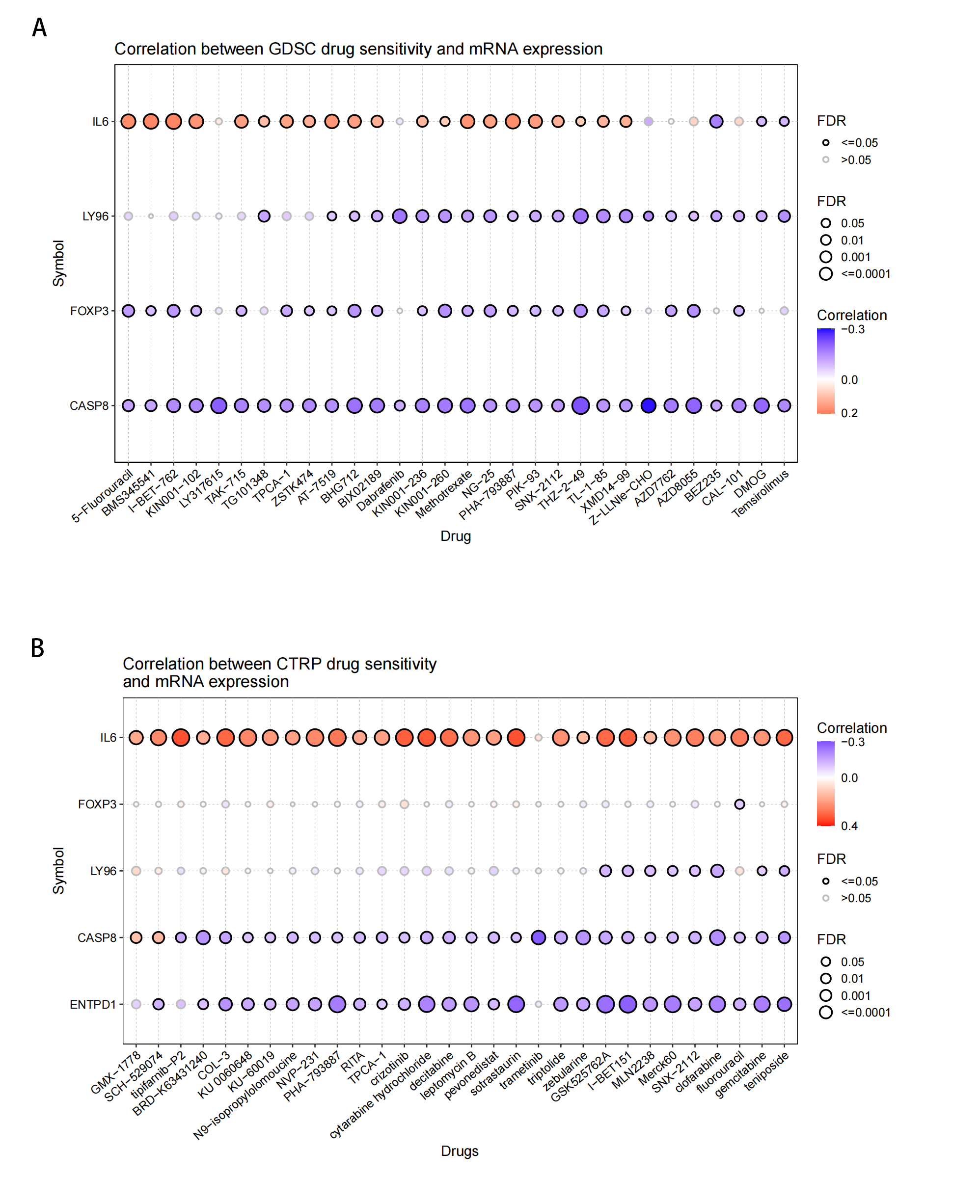

Supplement: Supplementary Figure 6 — The correlation between the expression level of ENTPD1, CASP8, LY96, FOXP3, and IL6 and drug sensitivity in pan-cancer through the GSCA database. [file Image_6.tif]
